# Breaking barriers: differential service deliveries for community HIV/AIDS services action and response in Northern Nigeria

**DOI:** 10.1186/s12889-026-27087-9

**Published:** 2026-03-23

**Authors:** Zayyanu Shitu, Zaynab U Nyako, Hamza Musa Dahiru, David Olusegun Oyedeji, Wole Fajemisin, Abdulsamad Salihu, Jennifer Anyati

**Affiliations:** 1 MEBS Global Reach Nigeria, Abuja, Nigeria; 2 Society for Family Health, Abuja, Nigeria

**Keywords:** Key Population, Differential Service Delivery model, One Stop Shop, Community Clinical Assistant Point

## Abstract

**Introduction:**

Human Immunodeficiency Virus (HIV) reported alarmingly increase daily, and poses threat to epidemic control globally. A significant number of these new cases (up to 70%) is being accounted for by the Key Population (KP). However, stigma and discrimination in our conventional healthcare systems lead to poor access to treatment among these populations. The current study reviews some of the healthcare models implemented for KP HIV program.

**Method:**

A retrospective data review of KP patients accessing care using the One Stop Shop (OSS) and Community Clinical Service Assistant Points (CCSAPs) models for HIV care in Zamfara State, Nigeria (May 2022 to May 2023). Important patient data were extracted, analysed and explored in frequency and percentage. Pearson’s chi-square and fisher exact test were also used.

**Results:**

Of the 4043 patients, 3737 patients with known viral load were used, CCSAPs had (77.8%) patients and OSS (22.2%). The majority were females (60.7%). The ages were between 25 and 34 years of ages (42.9%). The patients had a mean weight of 58.15Kg. Overal retention to care was (98.6%) with suppression rate of (99.4%), with CCSAPs (99.6%) suppression rate and OSS (99.0%) suppression.

**Conclusion:**

Both the CCSAPs and OSS models demonstrated a high rate of viral load suppression and retention in care for KP.The CCSAPs particularly excelled in viral suppression rates and patient retention. More HIV programs need to adopt these service delivery to improve access to care and sustain the progress for epidemic control.

## Introduction

Human Immune-Deficiency Virus (HIV) remains an alarming public health threat globally with 4000 new infections every day as reported in 2021 [1]. According to UNAIDS, about 38.4 million people are living with the virus globally, out of which 1.8 million are in Nigeria [2]. These numbers amounts to over 600,000 deaths in the year 2024 alone [3]. Key population (KP) comprising of Men who have sex with men (MSM), female sex workers (FSW), persons who inject drugs (PWID), transgenders, prisoners and their partners, accounted for 70% of new infections in recent times [1]. Due to stigma and discriminations in our conventional healthcare structure, these populations experience barriers to accessing adequate HIV testing services and treatments and care which result in a failed epidemic control globally [4, 5]. According to National Agency for control AIDS (NACA) in the year 2020, Major barriers hindering KP access to HIV services in conventional healthcare settings include stigma and discrimination from healthcare providers, breach of confidentiality, fear of legal repercussions, lack of KP-friendly services, and limited structural support, all of which discourage care-seeking and impede treatment outcomes [6].

Despite countless efforts to improve healthcare access and services to mitigate HIV/AIDS among KPs in Nigeria, the conventional facility-based service delivery continues to hinder these populations from getting quality care [7]. However, this is not the case for the broader, low HIV-rate general population. Nigeria HIV/AIDS indicator Survey shows HIV prevalence among adult in stands at 1.4%, with 1.8% of which are women and 1.0% are men. Annually, approximately 8 new HIV cases are reported per 10,000 adults between the ages 15–64 years, However, the highest incidence rate is with ages 25–34. In Nigeria’s household, about 3.1% them have at least one HIV case, with a higher frequency in rural households (3.3%) compared to urban households with (2.8%) [8]. The World Health Organization indicates high-risk groups of individuals face greater HIV risks, with FSW at 10 times more risk than the general population, MSM at 24 times, transgender women at 49 times more risk, and PWIDs at 28 times the risk [9]. This highlights the need for targeted programs to address these disproportions.

The World Health Organisation and other global partners recommended Differential Service Delivery (DSD) models through “differentiated care models” to improve testing services, treatment outcomes, and retention to care for all people living with HIV [10, 11]. Some of the DSD models are targeted towards KPs and they include the One Stop Shop (OSS) which integrates multiple service delivery including testing, counseling, treatment, and retention and other important intervention within a single location. The Community Clinical Service Assistant Points (CCSAPs) model involves leveraging on community-based healthcare facilities to provide similar services. Other models include home visits and telemedicine and digital health solutions which involves the use to technologies to provide consultations, care, and support to the KPs, reducing the need for physical appearance [7].

The current study is a programmatic review that evaluates and describes the OSS as well as the CCSAP differential service delivery models for HIV care in Key Population community HIV services for action and response, their effectiveness in the quality of HIV viral load, access, and retention to care. Additionally, the study explores potential effectiveness of these models overall HIV programs service delivery improvement.

## Method

### Study design

This study employed a retrospective descriptive programmatic description using routinely collected aggregate data from the Society for Family Health’s program data. The analysis descriptively showed how effective the two differentiated service delivery (DSD) models are for key populations: the One Stop Shop (OSS) model and the Community Clinical Service Assistant Points (CCSAPs) model.

The current study was not designed to establish relationships or comparative effectiveness. Rather, it descriptively characterizes service delivery patterns and observed outcomes within OSS and CSAP models. The analysis was guided by a conceptual framework that recognizes that service delivery outcomes are shaped by differences in service delivery context and processes across models, without implying causal or comparative inference: OSS deliveres its HIV/AIDS services in a single facility in the city capital, while CCSAP delivers decentralized, community-based services intended to increase accessibility, retention, and client engagement at different geographical locations.

### Study location and setting

The study was conducted in Zamfara State, Northwestern Nigeria, with a population of over 5 million people [12]. The state is predominantly rural, with agriculture and livestock farming as the main sources of livelihood. Socioeconomic indicators are low compared to national averages, with poverty and limited access to healthcare services serving as major challenges. Data were collected from 14 Local Government Areas (LGAs), including Zurmi, Maradun, Talata Mafara, Gusau, Kaura Namoda, Bungudu, Chafe, Maru, Anka, Bukkuyum, Gummi, Bakura, Birnin Magaji, and Shinkafi.

### Description of service delivery models

We analysed data obtained from the One Stop Shop (OSS) and the Community Clinical Service Assistant Points (CCSAPs).

One-Stop Shop (OSS): This model is located in Gusau, the administrative capital of Zamfara State. It is a comprehensive clinic providing integrated services exclusively for Key Populations. Facilities include clinical consultation, laboratory services, pharmacy, cervical cancer screening, a data entry room, and adherence counseling. The OSS is staffed with a multidisciplinary team including a medical practitioner (clinical supervisor), pharmacist, laboratory scientist, nurse, adherence counselor, and front-desk officer. It offers HIV, STI, and mental health care, plus linkages and referrals. OSS Model started in late year 2021,

Community Clinical Service Assistant Points (CCSAPs): These are decentralized service points located in the rural districts across 13 local governments. They involve a variety of facility types (government primary healthcare centres, general hospitals, and private care centres) that have been approved and supported by the OSS. CCSAPs replicate many of the HIV-care services offered at the OSS, adapted to community settings to improve access, reduce travel/time barriers, and address stigma for clients. CCSAP model of care was introduced into the program in early 2022.

### Study population and sampling

The study employed a purpose sampling technique, we included key populations (MSM, FSW, PWID, transgender persons, prisoners and people in confined settings) as well as their sexual partners, and children of KPs receiving ART at OSS and CCSAPs between May 2022 and May 2023. This is as categorised by UNAIDS [13].

A purposive complete enumeration approach was applied, whereby all OSS and CCSAP clients enrolled during the study period were considered. Analyses of viral load suppression were restricted to clients with available VL results. This represents a review of all eligible program data rather than a true census, and we acknowledge that excluding clients without VL results may introduce bias.

### Inclusion criteria

The current study included records of HIV/AIDS positive KPs, their sexual partners and children of KPs, within the study location who are accessing care in the OSS or CCSAPs supported by the program. The study excluded those patients who have not received their viral load results at the time of data collation.

### Data source, variables

Demographic and ART-related data of clients attending OSS and CCSAPs were abstracted from preexisting database entered into the Lafiya Management Information System (LAMIS) from client care cards. The data between May 2022 to May 2023 were retrieved as a Microsoft Excel sheet via the Retention Audit and Determination Tool (RADET). LAMIS is a nationally approved electronic medical record system used in Nigeria for HIV program monitoring [14]. The Retention and Audit Determination Tool (RADET) is a standardized reporting template used to extract and validate program data on client treatment outcomes [15] These systems have an in-built validation checks, also the programs consucts routine reviews, data quality audits to enhance completeness and consistency. Despite these processes, the exported data were further cleansed to adress reporting errors, and incomplete documentation, which may affect reliability.

Data obtained described the gender, target group, weight, age, viral load suppression, DSD model/enrolment setting, and antiretroviral therapy. In addition to the retrieved data. Data on patient interruption to care, discontinuation of ART, transfer out, mortality, currently active on ART, and reinitiated on ART of care were retrieved. All data were harmonised and cleansed into a single sheet. Cleansed data were uploaded into the International Business Machines Corporation, Statistical Package for Social Sciences (IBM SPSS Version 24) for analysis. Missing data were minimal for key variables due to use of routine program records; no imputation was performed.

### Definitions

#### Variables define according to national agency for control of aids national hiv and aids strategic framework 2021–2025 [16]

1. Active on ART-care: Clients who are alive and currently receiving antiretroviral therapy at the reporting site during the review period, it is retention in care in the current study.
2. Lost to Follow-Up (LTFU): Clients who have not had any clinical contact or drug pick-up for ≥ 90 days from their last expected clinic visit.
3. Transfer Out: Clients formally referred and documented to have moved their care to another ART facility.
4. ART Discontinuation: Clients whose treatment was stopped permanently due to clinical decision, client’s choice, or other programmatic reasons.
5. ART Reinitiation: Clients who previously discontinued or were LTFU but returned and restarted ART.
6. Viral Load Suppression: An HIV-positive client on ART with a viral load result < 1000 copies/ml at the time of measurement.
7. Viral Load Non-Suppression: An HIV-positive client on ART with a viral load ≥ 1000 copies/ml at the time of measurement [17].
8. Access to Care: Access to care in HIV refers to the ability of individuals to identify their health needs, seek appropriate HIV-related health services (such as testing, linkage to care, antiretroviral therapy, and support), reach those services physically and economically, and obtain continuous, appropriate care that meets their needs across the HIV care continuum. This concept incorporates dimensions such as availability, acceptability, affordability, and appropriateness of services, recognizing the role of both demand and supply factors in enabling people to receive care [17].
9. Previous ART status: This describes the KP’s individual’s history of exposure to antiretroviral therapy before the current episode of HIV care [18].
10. current ART status: This indicates the individual’s treatment state at a specific point in time, hether they are actively receiving ART, newly initiated, interrupted, or not on treatment [18].

### Statistical analysis

Descriptive statistics were used to summarize demographic and clinical characteristics of clients within each DSD model. Pearson’s chi-square tests and Fisher’s Exact Test were used *exploratorily* to describe differences in the distribution of selected variables across models; results are not interpreted as evidence of association or comparative effectiveness.

## Result

Between May 2022 and May 2023, a total of 4,043 people living with HIV accessed antiretroviral therapy and other services using the two differentiated service delivery (DSD) models, OSS and CCSAP. Of these, 904 clients received care through OSS, while the remaining 3,139 were enrolled in CCSAP. For the purpose of this study, our data analyses were focused on 3,737 clients whose viral load results were available by May 2023.

Regarding demographic characteristics, a larger proportion of patients were female (2,269; 60.7%) compared with male patients (1,468; 39.3%). Age distribution showed that the majority of patients were between the ages of 25 and 34 years (1,602; 42.9%), followed by those aged 35 to 44 years (1,002; 26.8%). The youngest age group, children under 14 years, represented a small proportion of clients (35; 0.9%), and older adults aged 55 years and above accounted for 90 clients (2.4%). The mean weight of clients across both models was 58.15 kg (Table 1).

**Fig. 1:**
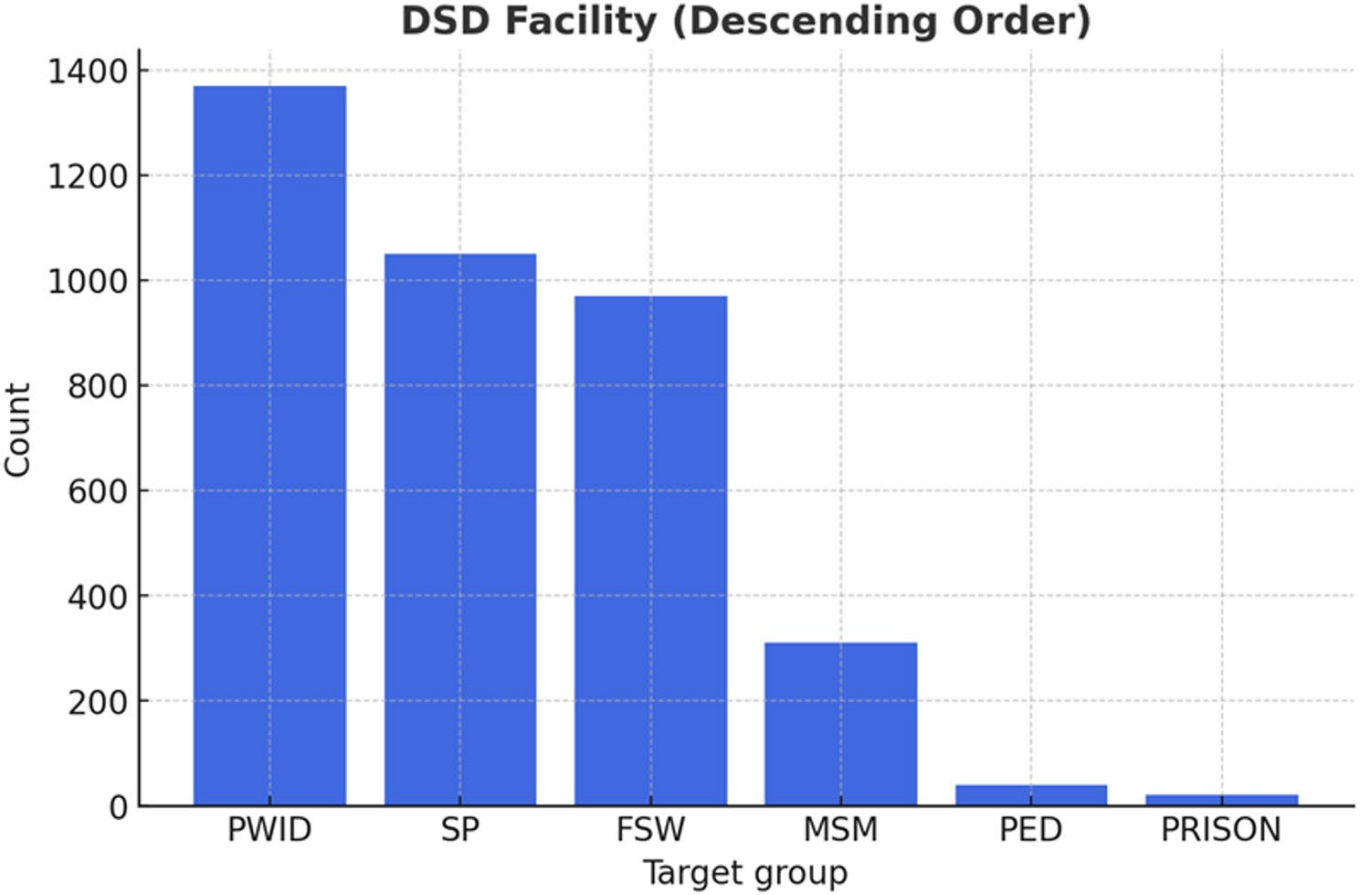
Graphical distribution of key population target groups in care (May 2022- May 2023)

In terms of service delivery engagement, 2,907 patients (77.8%) received care through CCSAP, whereas 830 (22.2%) were enrolled in OSS. Patients identified as people who inject drugs (PWIDs) formed the largest subgroup accessing services (1,377; 36.8%), followed by sexual partners of female sex workers and men who have sex with men (1,040; 27.8%). Other groups, such as prisoners (14; 0.4%) and children of key populations (36; 1.0%), represented the smallest proportions of patients accessing services (Fig. 1). These observations provide an overview of the distribution of patients across key target populations and service delivery contexts.

Across both DSD models, 3,716 patients (99.4%) achieved viral load suppression, while 21 clients (0.6%) had unsuppressed viral loads. Regarding antiretroviral therapy status, the vast majority of clients were current active ART care (3,694; 98.6%). Smaller numbers of patients had transferred out to other facilities (23; 0.6%), experienced mortality (3; 0.1%), interrupted or stopped treatment (07; 0.2%), or restarted treatment after interruption (9; 0.2%).

When disaggregated by service delivery model, outcomes such as viral suppression, ART status, mortality, treatment interruptions, and transfers are presented separately for OSS and CCSAP, (Table 2). The distribution of viral suppression status appeared similar across OSS and CSAP models (χ² test, *p* = 0.42). In contrast, the distribution of current antiretroviral therapy regimens differed across models (χ² test, *p* < 0.005). These findings are presented descriptively and are not intended to imply comparative effectiveness.

**Table 1:** Demographic profile and clinical characteristics of KPs (*n* = 3737), May 2023-May 2024 in Zamfara State, Nigeria

Variable	Category		*n* (%)	Total (*N*)	Mean (SD)
Gender	Male		1468 (39.3)		
	Female		2269 (60.7)	3737	
Age (years)	< 14		35 (0.9)		
	15–24		584 (15.6)		
	25–34		1602 (42.9)		
	35–44		1002 (26.8)		
	45–54		424 (11.3)		
	≥ 55		90 (2.4)	3737	
Weight (kg)	—	—		3737	58.15 (7.82)
DSD Facility	OSS	830 (22.2)			
	CCSAP	2907 (77.8)		3737	
KP Typology	FSW	964 (25.8)			
	MSM	306 (8.2)			
	PWID	1377 (36.8)			
	Prison	14 (0.4)			
	SP	1040 (27.8)			
	cKPs	36 (1.0)		3737	
Months on ART	6 months	3713 (99.4)			
	4 months	2 (0.1)			
	3 months	18 (0.5)			
	1 month	1 (0.0)		3737	
	0.5 month	3 (0.1)			
Viral Load Status	Suppressed	3716 (99.4)			
	Unsuppressed	21 (0.6)		3737	
Previous ART Status	Active	3694 (98.8)			
	Death	3 (0.1)			
	IIT	10 (0.3)			
	Stopped treatment	7 (0.2)			
	Transfer out	23 (0.6)		3737	
Current ART Status	Active	3685 (98.6)			
	Active restart	9 (0.2)			
	Death	4 (0.1)			
	IIT	4 (0.1)			
	Stopped treatment	7 (0.2)			
	Transfer out	28 (0.7)		3737	

*Descriptive statistics, frequencies and percentages, Mean (Standard deviation),

*Children of KPs(cKPs)

*Sexual Partners (SP)

^*^denominators vary due to missing data

Table 2 presents the distribution of participants’ demographic and clinical characteristics across the two differentiated service delivery (DSD) models using row percentages. Most participants were enrolled in the CCSAP model across all categories. The distribution of sex did not differ meaningfully between models (*p* = 0.106). Differences were observed in the age distribution of participants across DSD models, with higher proportions of older age groups enrolled in CCSAP compared with OSS (*p* = 0.005). Similarly, the distribution of key population types varied across models (*p* < 0.001), reflecting differences in service utilization patterns among specific groups. Viral load suppression was high in both models, with over 99% of participants virally suppressed in each group. Due to the extreme imbalance in viral load categories, Fisher’s Exact Test was applied, and no meaningful difference in viral suppression distribution between models was observed (*p* = 0.21). For ART status, outcomes were summarized as active versus non-active due to small numbers in individual non-active categories. Using Fisher’s Exact Test, no meaningful differences were observed between CCSAP and OSS for current ART status (*p* = 0.31) or previous ART status (*p* = 0.18). These findings are presented descriptively and should be interpreted as exploratory.

**Table 2:** Distribution of participant characteristics within OSS and CCSAP models (*n* = 3,737)

Variable	Category	CCSAP *n* (%)	OSS *n* (%)	Test	*p*-value
Sex	Female	1745 (76.9)	524 (23.1)	Chi-square	0.106
	Male	1162 (79.2)	306 (20.8)		
Age (years)	< 14	26 (74.3)	9 (25.7)	Chi-square	0.005
	15–24	439 (75.2)	145 (24.8)		
	25–34	1218 (76.0)	384 (24.0)		
	35–44	804 (80.2)	198 (19.8)		
	45–55	340 (80.2)	84 (19.8)		
	≥ 55	80 (88.9)	10 (11.1)		
Key population type	FSW	766 (79.5)	198 (20.5)	Chi-square	< 0.001
	MSM	203 (66.3)	103 (33.7)		
	PED	26 (72.2)	10 (27.8)		
	Prison	3 (21.4)	11 (78.6)		
	PWID	1138 (82.6)	239 (17.4)		
	SP	771 (74.1)	269 (25.9)		
Viral status	Suppressed	2894 (99.6)	822 (99.0)	Fisher’s Exact	0.21
	Unsuppressed	13 (0.4)	8 (1.0)		
Current ART status	Active	2872 (77.9)	813 (22.1)	Fisher’s Exact	0.31
	Non-active	35 (70.0)	15 (30.0)		
Previous ART status	Active	2881 (78.0)	813 (22.0)	Fisher’s Exact	0.18
	Non-active	26 (65.0)	14 (35.0)		

*n*= number, *DF*= degree of freedom

Non-active includes death, interruption in treatment (IIT), stopped treatment, and transfer out

Percentages are row percentages, representing the proportion of participants within each category who are in each DSD model

Fisher’s Exact Test was used for variables with small expected cell counts; Pearson’s chi-square test was used for all other variables

All analyses are exploratory and descriptive, not intended to establish causal associations

**Fig. 2:**
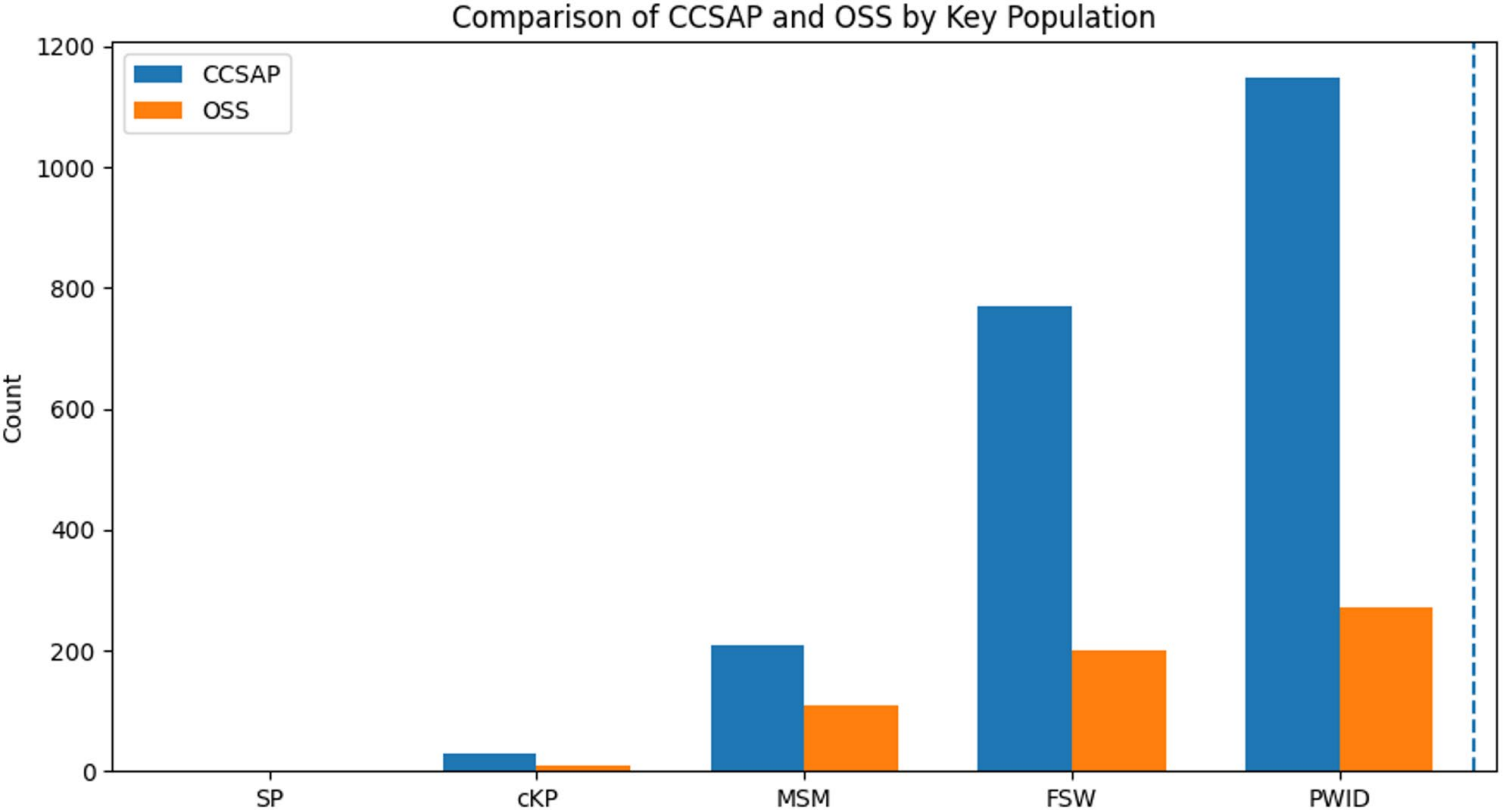
Facility and Key Population Distribution

Figure 2 illustrates the distribution of key population groups across two types of facilities (CCSAP and OSS). Most FSW, PWID, and SP clients were served by CCSAP, with PWID being the largest group overall. On the other hand, OSS facilities had relatively fewer clients overall but proportionally more prisoners, MSM, and SP compared to CCSAP.

## Discussion

The gender distribution showing a higher number of females accessing care 2269 (60.7%) conforms with the global epidemiology distribution, where women are the most affected by the virus [11]. This is applicable in this study context because key population data were also defined by their gender. The adult age group between 25 and 34 years shows higher prevalence, which aligns with many regions around the world. However, it is noteworthy that, during the sampled period, children (< 14 years) and elderly patients (> 55 years) were poorly represented for care, which is in line with a study reported in North and East Africa [19–21]. These children of KPs were misrepresented because KPs hardly move around with children due to the nature of their business, similarly the MSM group present little to no children among them. Moreover, elderly KPs are usually out of the circle, therefor they were naturally represented in few numbers very. In addition to that, the current program doesn’t offer Prevention of Mother-to-Child Transmission (PMTCT) services, hence children of KP are misrepresented. There is an observed difference in access to care by the KP between the two DSD models, with CCSAPs having a larger percentage of patients 2907 (77.8%) compared to OSS 830 (22.2%). The preference for CCSAPs might be attributed to their easy access, proximity to hotspot communities, and comprehensive approach to patient care, which likely attracts more patients seeking universal care [22, 23]. In addition, the CCSAPs are equipped and supported by the program with the necessary training, close monitoring and evaluation, and tools for quality services. Within the CCSAPs model, the KPs were represented well, and the PWIDs and Sexual Partners of FSW and MSM accessed the care most, demonstrating the success of the targeted KP intervention program and showcasing an excellent viral load supression.

An incredible finding was the program combined the DSD model’s high viral load suppression rate 3716 (99.4%). This is a noteworthy programmatic achievement, showing the effectiveness of first-line antiretroviral drug therapy and the quality of adherence counselling in controlling viral replication and decreasing the risk of transmission. Furthermore, the very inconsequential interruption to care (IIT) of 0.3% could be a positive factor, showcasing the models potentials in KP patients retention to HIV care. This outcome is in line with what was previously reported in sub-Saharan Africa and Asia [24–26]. The high viral suppression rate reveals the overall quality of HIV care provided by the employed DSD models. Notwithstanding, for the overall high viral suppression rate, there are interesting differences to note between the two models. A slightly higher viral load suppression rate was recorded at CCSAPs 99.6% compared to OSS 99.0%. This difference, although small, might possess clinical implications which require additional investigation. The difference could be due to the demograohic distribution of KPs across the state, making more economical to access care at CCSAPs, and hence lower IIT and and higher viral suppression. It is important to study the contributing factors, including the best practices employed leading to higher viral suppression rates in CCSAPs, which could be potentially replicated in other settings.

There are also disparities in patient retention to care and outcomes among the service models to the HIV care. The CCSAPs had a greater proportion of patients actively in care 2872 (77.9%) compared to OSS 813(22.1%), representing better retention in the comprehensive care model. This is good for program sustainability, Among the 3,737 participants, there were 4 deaths (0.1%) and 4 instances of treatment interruption (0.1%) across both DSD models. Due to the very small number of events, these outcomes are reported descriptively and no inference regarding differences between CCSAP and OSS is made.

No clear variation was observed between the two DSD models with respect to viral load suppression rates. In contrast, differences were observed in the distribution of current antiretroviral therapy (ART) regimens across the models. These observations reflect variation in treatment approaches and management practices within the two service delivery models, which may influence how care is provided across different contexts. Further exploration of these patterns may help clarify their programmatic relevance and inform optimization of HIV service delivery across models.

The observed patterns in facility choice for HIV/AIDS care between CCSAP and OSS reflect differences in operational characteristics and client accessibility factors that shape service delivery for key populations in Nigeria and Zamfara State. The greater representation of younger participants (< 35 years) within OSS aligns with evidence suggesting that younger key populations tend to utilize centralized, youth-friendly services offering comprehensive care [27]. The high representation of prisoners within OSS 11 (78.6%) is consistent with structural constraints faced by this population, including restricted mobility and the need for coordinated healthcare services, which are more readily addressed through integrated facility-based models [28]. Similarly, the substantial representation of PWID 1136 (82.6%) and FSW 766 (79.5%) within CCSAPs is consistent with findings from sub-Saharan Africa indicating that community-based HIV service delivery models can reduce stigma, transportation costs, and time-related barriers key considerations for marginalized populations navigating criminalized or stigmatized identities [29]. In the context of Zamfara State and other parts of Nigeria, where key populations face economic and social barriers to healthcare access, the integration of CCSAPs within community health structures may enhance confidentiality and reduce experiences of discrimination compared with conventional facility-based services [30].

The pattern for treatment history reveals some important HIV programmatic inferences for retention and continuity of care. The higher representation of treatment interrupters 4 (57.1%), transfer-outs 11 (39.3%), and short-duration patients (0.5–4 months) at the OSS model indicates that it serves as a critical re-engagement point for individuals with complex opportunistic infections. This pattern aligns with studies demonstrating that centralized healthcare facilities with multidisciplinary teams are better equipped to address multifaceted healthcare challenges such as adherence difficulties, mental health comorbidities, and social instability that drive treatment discontinuation among KPs [31, 32]. The concentration of clients with interrupted treatment histories at OSS suggests this model functions as a safety net for individuals who have cycled out of care, offering specialized services that may not be readily available in decentralized settings. Furthermore, the OSS’s establishment before the CCSAP may also explain its role as the primary entry point for clients before decentralization expanded access, creating a historical pattern of initial engagement that persists even as alternative models emerge.

The presence of clients with complex treatment histories at OSS may also reflect the reality that key populations often face unique barriers to sustained engagement, including mobility, criminalization, stigma, and unstable living conditions, all of which contribute to interrupted care trajectories. Centralized models like OSS can provide a consistent point of contact and comprehensive services that address these layered vulnerabilities. However, the predominance of stable, longer-term patients at CCSAPs evident from lower proportions of treatment interruption validates the model’s effectiveness in maintaining continuity of care in Nigeria [3, 33]. This finding suggests that once clients are successfully engaged and stabilized, decentralized community-based models offer accessibility and convenience that support long-term retention. The community integration inherent in CCSAPs may reduce barriers related to distance, transportation costs, and visibility that can deter KPs from accessing care at more centralized facilities.

These findings highlight a complementary balance in the roles of both DSD models: OSS appears most effective as a specialized hub for managing KP clients with complex cases and serving as a critical point of entry or re-entry to care, while CCSAPs provide more sustainable, accessible care that reduces loss to follow-up for stable clients. This differentiated approach suggests that rather than viewing these models as competing alternatives, they may be optimally deployed as part of an integrated continuum of care. Such a continuum would leverage the strengths of each model—centralized expertise and comprehensive services at OSS for clients requiring intensive support, and decentralized accessibility and community embeddedness at CCSAPs for maintenance of stable clients. This layered service delivery framework could enhance overall program effectiveness by matching client needs with appropriate service intensity and location.

This study is among the few that have examined differential healthcare models among Key Populations in Nigeria, employing a large sample size of real-world data from the HIV KP program. To our knowledge, it is among the few studies that compare important components of DSD models alongside clinical and sociodemographic characteristics of KPs, providing valuable insights into how different service delivery approaches may serve distinct client profiles and needs within the same population. The descriptive and exploratory nature of this analysis allows for a nuanced understanding of patterns and trends without overstating causal relationships, which is particularly valuable in the context of program implementation where multiple factors interact in complex ways.

Some of the limitations of this study relate to the study design. The study is a retrospective data review that could have an impact on data accuracy, as it relies on documentation quality and completeness in routine programmatic records. Data entry errors, missing information, and inconsistencies in record-keeping practices across sites may introduce measurement error. Similarly, there might be selection bias in how KPs access care, which can influence the generalizability of findings. Clients who choose or are able to access OSS versus CCSAP may differ in ways that are not fully captured in available data such as proximity to facilities, social networks, personal preferences, severity of illness at presentation, or levels of social support and these unmeasured differences could confound observed patterns. The study did not assess all factors that could contribute to access and quality of HIV care by KPs, including structural determinants such as legal status of key population activities, policing practices, housing stability, and economic security. The study excluded clients whose VL results were not received at the time of the study; even though this might have an impact on the findings, the proportion excluded was small and unlikely to significantly alter the overall patterns observed.

Moreover, the scope of this study is restricted to variables available in routine program data, which were limited to demographic and clinical indicators. Behavioral and stigma-related variables, which are key to understanding KP service utilization, were not captured within these routine systems. Factors such as experiences of discrimination within healthcare settings, internalized stigma, disclosure concerns, substance use patterns, violence exposure, and peer support networks are all known to influence engagement and retention among KPs but were not available for analysis in this dataset. Therefore, the study does not fully reflect the broader structural, behavioral, and psychosocial barriers faced by key populations, and the patterns observed should be interpreted in light of these unmeasured influences.

Similarly, the two DSD models differed at baseline in setting, client characteristics, and service delivery approaches, representing naturally occurring variation in program implementation rather than randomized assignment. Regression models could not be fitted due to the highly imbalanced outcome (few unsuppressed cases), so descriptive statistics employing chi-square tests were used instead. While these provide valid comparisons of distributions and patterns, they do not adjust for baseline differences between the groups, and therefore causality cannot be inferred. Results were interpreted in an exploratory and descriptive manner only, focusing on identifying patterns and generating hypotheses rather than establishing definitive conclusions about model superiority or effectiveness. This approach is appropriate given the observational nature of the data and the multiple confounding factors that may influence outcomes in real-world program settings.

Thus, careful consideration of these strengths and limitations is crucial when interpreting and applying the study discoveries to clinical practice, programs, or policy decisions. The findings should be viewed as hypothesis-generating and as providing preliminary evidence that can inform program planning and service delivery strategies, while recognizing the need for more rigorous evaluation designs—such as prospective cohort studies or implementation science approaches—to establish causal relationships and test specific interventions.

## Conclusion

The current study delivers valuable evidence on the performance of two DSD models employed programmatically for ART delivery in HIV care among key populations. The high viral load suppression rates observed in both models are highly commendable and reflect substantial success in achieving virological control through ART, demonstrating that differentiated service delivery approaches can effectively support treatment adherence and clinical outcomes when tailored to the needs of key populations. These findings contribute to the growing body of evidence supporting the feasibility and effectiveness of diverse service delivery strategies in resource-limited settings, particularly for populations facing significant barriers to healthcare access.

The study also underscores the critical importance of KP-targeted interventions, with both OSS and CCSAPs demonstrating capacity to engage and retain key populations in care a population historically underserved and marginalized within health systems. The success of these models in achieving high viral suppression rates among key populations is particularly noteworthy given the multiple structural, social, and legal barriers this population faces, including criminalization, stigma, discrimination, violence, and social exclusion. These results suggest that when services are designed with key population needs in mind—incorporating elements such as non-judgmental care, confidentiality, flexible scheduling, peer support, and linkages to comprehensive services—substantial improvements in health outcomes are achievable.

However, the study reveals important variations in access to care, retention patterns, and client characteristics between the two models, which merit further optimization and investigation. The OSS model appears to serve a distinct role as a specialized hub for clients with complex treatment histories, those newly entering care, and individuals requiring intensive support or management of opportunistic infections. In contrast, CCSAPs demonstrate strengths in maintaining stable clients in long-term care through decentralized, community-based service delivery that enhances accessibility and reduces barriers to sustained engagement. These complementary patterns suggest that rather than representing competing alternatives, these models may function most effectively as part of an integrated continuum of care, with clients transitioning between models based on clinical status, stability, and individual needs.

The exploratory and descriptive nature of this analysis has identified important patterns that warrant further investigation through more rigorous study designs. Future research should employ prospective cohort studies, mixed-methods approaches, and implementation science frameworks to better understand the mechanisms through which each model achieves outcomes, the factors influencing client selection and retention in each setting, and the optimal pathways for integrating these models within comprehensive HIV care systems. Additionally, future studies should incorporate behavioral, psychosocial, and structural variables including stigma experiences, legal environment, social support, and economic stability to provide a more complete understanding of the factors shaping key population engagement with HIV services.

By carefully understanding the strengths and limitations of each model, alongside the distinct roles they play in the care continuum, policymakers, programmers, and healthcare providers can make evidence-informed decisions to optimize HIV care delivery and accomplish better outcomes for patients, especially among key populations. Strategic investments should consider how to leverage the specialized capabilities of centralized models like OSS while simultaneously expanding the reach and sustainability of community-based approaches like CCSAPs. Such differentiated yet integrated approaches hold promise for achieving the UNAIDS 95-95-95 targets among key populations, reducing health disparities, and ultimately contributing to epidemic control. The findings from this study provide a foundation for ongoing program improvement, strategic planning, and policy development aimed at ensuring that all key populations have access to high-quality, acceptable, and effective HIV care regardless of their clinical complexity or social circumstances.

## Data Availability

The data set generated during/or analyzed during the current research study are available with the corresponding author on reasonable request.

## References

[1] WHO. WHO recommends long-acting cabotegravir for HIV Prevention. Retrieved August 5th, from 2022. https://www.who.int/news/item/28-07-2022-who-recommends-long-acting-cabotegravir-for-hiv-prevention.

[2] UNAIDS. Nigeria. Retrieved August, 17th from 2021. https://www.unaids.org/en/regionscountries/countries/nigeria.

[3] Lo, J., Nwafor, S. U., Schwitters, A. M., Mitchell, A., Sebastian, V., Stafford, K.A., … McIntyre, A. F. Key population hotspots in Nigeria for targeted HIV program planning: mapping, validation, and reconciliation. JMIR Public Health and Surveillance, 2021;7(2):e25623. 10.2196/25623. 10.2196/25623 PMC7939933 33616537

[4] Djomand G, Quaye S, Sullivan PS. HIV epidemic among key populations in west Africa. Curr Opin HIV AIDS. 2014;9(5):506. 10.1097/COH.0000000000000090. 25010898 10.1097/COH.0000000000000090 PMC4804351

[5] Macdonald V, Verster A, Baggaley R. A call for differentiated approaches to delivering HIV services to key populations. J Int AIDS Soc. 2017;20:21658. 10.7448/IAS.20.5.21658. 28770592 10.7448/IAS.20.5.21658 PMC5577716

[6] Nigeria HIV/AIDS indicator Survey NAIIS. Retrieved, October 17th. from 2018. https://www.naiis.ng/.

[7] World Health Organisation. WHO publishes new guidelines on HIV, hepatitis and STIs for key populations. Retrieved October 17th, From 2022. https://www.who.int/news/item/29-07-2022-who-publishes-new-guidelines-on-hiv--hepatitis-and-sti-for-key-populations.

[8] World Health Organization. Consolidated guidelines on the use of antiretroviral drugs for treating and preventing HIV infection: recommendations for a public health approach. 2nd ed. Geneva: World Health Org. 2016. Available from: https://www.who.int/publications/i/item/9789241549684. 27466667

[9] The Global Fund to Fight AIDS, Tuberculosis and Malaria. New toolkit for differentiated care in HIV and TB programs. 2015. Available from: https://www.theglobalfund.org/en/news/2015/2015-12-04-new-toolkit-for-differentiated-care-in-hiv-and-tb-programs/.

[10] Nigerian Investment Promotion Commission. Retrieved from 2023. https://www.nipc.gov.ng/nigeria-states/zamfara-state/.

[11] UNAIDS. Global HIV/AIDS Statistics, factsheet. 2023. https://www.unaids.org/en/resources/fact-sheet.

[12] Bekker LG. HIV control in young key populations in Africa. Lancet Child Adolesc Health. 2019;3(7):442–4. 31105054 10.1016/S2352-4642(19)30112-9

[13] Mumtaz, G. R., Chemaitelly, H., AlMukdad, S., Osman, A., Fahme, S., Rizk, N. A., …Abu-Raddad, L. J. (2022). Status of the HIV epidemic in key populations in the Middle East and north Africa: knowns and unknowns. The Lancet HIV, 9(7), e506-e516,doi.org/10.1016/S2352-3018(22)00093-5. 10.1016/S2352-3018(22)00093-5 35777412

[14] Shrestha R, Philip S, Shewade HD, Rawal B, Deuba K. Why don’t key populations access HIV testing and counselling centres in Nepal? Findings based on national surveillance survey. BMJ open. 2017;7(12). 10.1136/bmjopen-2017-017408. 10.1136/bmjopen-2017-017408 PMC5770839 29288177

[15] Macdonald V, Verster A, Baggaley R. A call for differentiated approaches to delivering HIV services to key populations. J Int AIDS Soc. 2017;20:21658. 10.7448/IAS.20.5.21658. 28770592 10.7448/IAS.20.5.21658 PMC5577716

[16] McCluskey SM, Pepperrell T, Hill A, Venter WD, Gupta RK, Siedner MJ. Adherence, resistance, and viral suppression on dolutegravir in sub-Saharan Africa: implications for the TLD era. Aids. 2021;35(Supplement 2):S127–35. 10.1097/QAD.0000000000003082. 34848579 10.1097/QAD.0000000000003082 PMC8647784

[17] Schramm, B., Temfack, E., Descamps, D., Nicholas, S., Peytavin, G., Bitilinyu-Bangoh,J. E., … Szumilin, E. (2022). Viral suppression and HIV-1 drug resistance 1 year after pragmatic transitioning to dolutegravir first-line therapy in Malawi: a prospective cohort study. The Lancet HIV, 9(8),. doi.org/10.1016/S2352-3018(22)00136-9. 10.1016/S2352-3018(22)00136-9 35905753

[18] Gangcuangco LMA. HIV crisis in the Philippines: urgent actions needed. Lancet Public Health. 2019;4(2):e84. 10.1016/S2468-2667(18)30265-2. 30738505 10.1016/S2468-2667(18)30265-2

[19] National Agency for the Control of AIDS (NACA). Nigeria People Living with HIV (PLHIV) Stigma Index 2.0 survey report. Abuja: National Agency for the Control of AIDS; 2021. Available from: https://naca.gov.ng/wp-content/uploads/2023/03/NIGERIA-PLHIV-STIGMA-INDEX-2.0-REPORT_2021.pdf.

[20] Idoko-Asuelimhen O, Alozie A, Iyamu I. Management and monitoring of PLHIV using electronic systems in Nigeria: Lafiya Management Information System (LAMIS). Abuja: Lafiya Management Information System (LAMIS); 2019.

[21] Aina M, Odeyemi K, Ologun O, Ntoimo L, Okonofua F. Patient and health facility attributes associated with skilled birth attendant utilization in Nigeria: A cross-sectional study. BMC Health Serv Res. 2022;22(1):1339.36368986

[22] Donabedian A. The quality of care: How can it be assessed? JAMA. 1988;260(12):1743–8. 10.1001/jama.260.12.1743. 3045356 10.1001/jama.260.12.1743

[23] UNAIDS. (n.d.). Key populations [web page]. Retrieved 1st. October, 2025 from https://www.unaids.org/en/topic/key-populations.

[24] Abodunrin OL, Bamidele JO, Olugbenga-Bello AI, Parakoyi DB. Preferred choice of health facilities for healthcare among adult residents in Ilorin metropolis, Kwara state, Nigeria. Int J health Res. 2010;3(2):79–86.

[25] Melvin AO. Prison health in Nigeria: A sociological discourse. Afr J Political Sci Int Relations. 2013;7(2):38–41.

[26] Grimsrud, A., Bygrave, H., Doherty, M., Ehrenkranz, P., Ellman, T., Ferris, R., …Bekker, L. G. Reimagining HIV service delivery: the role of differentiated care from prevention to suppression. Journal of the International AIDS Society, 2016;19(1),21484. 10.7448/IAS.19.1.21484 PMC5136137 27914186

[27] Bärnighausen T, Chaiyachati K, Chimbindi N, Peoples A, Haberer J, Newell ML. Interventions to increase antiretroviral adherence in sub-Saharan Africa: A systematic review of evaluation studies. Lancet Infect Dis. 2011;11(12):942–51. 10.1016/S1473-3099(11)70181-. 22030332 10.1016/S1473-3099(11)70181-5 PMC4250825

[28] Govindasamy D, Meher S, Ebrahim S, Gregson S, Richter L, Darbes L. Linkage to HIV care from a mobile testing unit in South Africa by different CD4 count strata. J Acquir Immune Defic Syndr. 2014;65(3):e13–4. 10.1097/QAI.0b013e31829a4aa4. 10.1097/QAI.0b013e31822e0c4c PMC3805962 21836524

[29] Eluwa GI, Adebajo SB, Eluwa T, Ogbanufe O, Ikpeme B, Ayenigbara O, Ahonsi B, Risher KA. Rising HIV prevalence among men who have sex with men in Nigeria: A trend analysis. BMC Public Health. 2015;15:1–8. 10.1186/s12889-015-2506-4. 31477073 10.1186/s12889-019-7540-4 PMC6721282

[30] oint United Nations Programme on HIV/AIDS. Global HIV & AIDS statistics — Fact sheet. UNAIDS. Retrieved January 5, 2026, from 2025. https://www.unaids.org/en/resources/fact-sheet.

[31] National Agency for the Control of AIDS. National HIV and AIDS strategic framework 2021–2025 (Final ed.). Federal Republic of Nigeria. 2021. https://naca.gov.ng/wp-content/uploads/2022/03/National-HIV-and-AIDS-Strategic-Framework-2021-2025-Final.pdf.

[32] Levesque J-F, Harris MF, Russell G. Access to health care: Conceptualising access at the interface of health systems and populations. BMC Public Health. 2013;13., Article 1. 10.1186/1471-2458-13-1. 10.1186/1475-9276-12-18 PMC3610159 23496984

[33] World Health Organization. Consolidated guidelines on the use of antiretroviral drugs for treating and preventing HIV infection (2nd ed.). World Health Organization. 2016. https://apps.who.int/iris/handle/10665/208825. 27466667

